# The role of perception and action on the use of allocentric information in a large-scale virtual environment

**DOI:** 10.1007/s00221-020-05839-2

**Published:** 2020-06-04

**Authors:** Harun Karimpur, Johannes Kurz, Katja Fiehler

**Affiliations:** 1 Experimental Psychology, Justus Liebig University Giessen, Giessen, Germany; 2 Center for Mind, Brain and Behavior (CMBB), University of Marburg and Justus Liebig University Giessen, Giessen, Germany; 3 NemoLab–Neuromotor Behavior Laboratory, Justus Liebig University Giessen, Giessen, Germany

**Keywords:** Spatial representation, Egocentric, Allocentric, Perception and action, Virtual reality

## Abstract

In everyday life, our brain constantly builds spatial representations of the objects surrounding us. Many studies have investigated the nature of these spatial representations. It is well established that we use allocentric information in real-time and memory-guided movements. Most studies relied on small-scale and static experiments, leaving it unclear whether similar paradigms yield the same results on a larger scale using dynamic objects. We created a virtual reality task that required participants to encode the landing position of a virtual ball thrown by an avatar. Encoding differed in the nature of the task in that it was either purely perceptual (“view where the ball landed while standing still”—Experiment 1) or involved an action (“intercept the ball with the foot just before it lands”—Experiment 2). After encoding, participants were asked to place a real ball at the remembered landing position in the virtual scene. In some trials, we subtly shifted either the thrower or the midfield line on a soccer field to manipulate allocentric coding of the ball’s landing position. In both experiments, we were able to replicate classic findings from small-scale experiments and to generalize these results to different encoding tasks (perception vs. action) and response modes (reaching vs. walking-and-placing). Moreover, we found that participants preferably encoded the ball relative to the thrower when they had to intercept the ball, suggesting that the use of allocentric information is determined by the encoding task by enhancing task-relevant allocentric information. Our findings indicate that results previously obtained from memory-guided reaching are not restricted to small-scale movements, but generalize to whole-body movements in large-scale dynamic scenes.

## Introduction

How is observing a ball thrown to you different from catching it? In the context of spatial coding, we want to understand whether our brain processes these two cases similarly. In everyday life, the world constantly provides us with opportunities to interact with objects. For successful human–object interaction, our brain needs to accurately and precisely locate the objects and build up stable spatial representations. Research in the field of spatial coding is concerned with the problem of how these spatial representations are computed and finally used for perceptual and action tasks.

The location of an object can typically be described by three points, one on each of the three axes that represent the Euclidian space. This naturally raises the question of where we place the origin of the three-dimensional coordinate system. The term reference frame refers to the nature of this origin and to where we embed the coordinate axes. Decades of studies provide evidence for a distinction between two classes of reference frames, namely egocentric and allocentric (Colby 1998; Klatzky 1998). In an egocentric reference frame, the location of an object is encoded relative to oneself, i.e., some part of the body. This plays a pivotal role when performing actions toward objects as we need to know, for example, how far we should reach. Compelling evidence demonstrates that action targets are coded in a gaze-centered (i.e., egocentric) reference frame (for an overview, see Crawford et al. 2011; Medendorp 2011). In an allocentric reference frame, we encode the location of an object relative to other objects, i.e., landmarks. Stable landmarks allow us to reliably compute spatial relations between objects. We therefore integrate allocentric information based on how reliable they are (Byrne and Crawford 2010) which affects late movement kinematics (Heath et al. 2007) and leads to an improved accuracy in online and delayed movement tasks (Krigolson and Heath 2004; Obhi and Goodale 2005).

A key method that has been used to test for allocentric (i.e., object-to-object) coding is the object-shift paradigm (Byrne and Crawford 2010). In such experiments, participants memorize a target object surrounded by other objects (landmarks). During a blank period, unbeknownst to the participant, the target disappears and the landmarks are subtly shifted. The participants’ task is to reach to the remembered location of the target object. The rationale is simple: There are two representations that can be accessed to solve this task. A) One could access an egocentric representation, where the target is coded relative to oneself (e.g., the body midline). If that is true, they should reach correctly without being affected by the shift of landmarks. B) One could access an allocentric representation, where the target is represented relative to landmarks in the surrounding of the target. If that is true, they should produce a systematic reaching error in the direction of where the landmarks were shifted. There is converging evidence from electrophysiological, imaging and behavioral research that egocentric and allocentric information are combined and weighted with respect to their reliability following a Bayesian account (Burgess 2006; Byrne and Crawford 2010; Chen and Crawford 2019; Committeri et al. 2004; Plank et al. 2010).

More recent memory-guided reaching experiments made use of the same rationale and investigated allocentric coding using more complex, naturalistic scenes instead of abstract stimuli. For example, in the breakfast scene experiments, participants freely encode a scene consisting of multiple objects placed on a table. After a brief mask and delay, the scene reappears with one random object missing (target) and the remaining objects (landmarks) either shifted or not. Participants then reached to the remembered location of the target on an empty scene. Reaching end points systematically deviated in the direction of object shift, suggesting the use of allocentric information. Allocentric coding was found to be facilitated when the objects were task relevant (Fiehler et al. 2014; Klinghammer et al. 2015), coherently shifted (Klinghammer et al. 2017) and semantically similar (Karimpur et al. 2019). While these studies advanced our understanding of potential factors facilitating allocentric coding, little is known about the generalizability of these results. The key issues we would like to address in this study concern: (i) space, (ii) scene dynamics, and (iii) task.

i. Space comprises more than just the environment in our immediate surroundings. However, visuomotor tasks that test allocentric coding usually required participants to point or reach to targets in close proximity. This raises the question of whether the results obtained in small-scale experiments (e.g., reaching to an object on the computer screen/table) generalize to large-scale environments. There is reason to believe that such a generalization occurs. For example, mental representations are built on the visually perceived space that can exceed the reachable space (e.g., by tool use, Berti and Frassinetti 2000; Longo and Lourenco 2006) and this seems to be especially important for establishing allocentric representations of large-scale spaces (Iachini et al. 2014). In support of that notion, many findings suggest that the action-relevant space scales contingent upon the situation and task at hand (action field theory; Bufacchi and Iannetti 2018). These findings motivated us to examine allocentric coding in spaces larger than previously examined in memory-guided reaching tasks. Virtual reality provides an excellent tool to study large-scale movements in real-world-like environments with full experimental control.
ii. Both the naturalistic images and the virtual environments that were previously used in memory-guided reaching tasks mentioned earlier relied on “still life”-like static scenes for which the encoding duration was rarely controlled for. Clearly, our everyday life is more dynamic, leaving it unclear if the findings could be replicated for brief and controlled presentation durations. This is especially important for real-time actions where there is little time to build up spatial representations of action targets.
iii. Previous experiments required participants to encode the static scene without performing an action (encoding-while-perceiving) and to use that representation to guide their actions later on (delayed action). Thus, it is unclear which representations are used to guide delayed actions for objects that were encoded as actual action targets (encoding-while-performing). Here, we would like to study the effect of task, perception vs. action, on allocentric coding of target locations. Performing an action inherently relies on an egocentric reference frame (e.g., Colby and Goldberg 1999) that probably leads to a mitigated use of allocentric information when an action is required or to a facilitation of allocentric coding when the performed task influences the weightings between landmarks. Related ideas were proposed and tested in human navigation research (e.g., Miller and Carlson 2011) where the term contextual salience has been used to describe that the landmark selection process does not only depend on perceptual salience, but also on the task (Caduff and Timpf 2008). For example, participants would choose landmarks based on where the landmarks were placed (e.g., at decision points) rather than how perceptually salient they are. Importantly, this was not found to be the case when switching from a navigation to a recognition task.

We addressed the three points described above (i to iii) by creating two virtual reality experiments on a soccer field (i) with participants situated in a throw-in scenario (ii) and landmarks (thrower vs. midfield line) systematically shifted after spatial encoding. The required response was to walk and place a ball at a remembered landing location as opposed to reaching it. Remembering the location requires successful encoding. The experiments differed in the task that had to be performed during spatial encoding (iii). In Experiment 1 (perceptual), we required participants to observe where the ball had landed. In Experiment 2 (action), we required participants to intercept the ball right before it would touch the ground and to remember the ball’s position at the moment of interception. The response required at the end of both experiments was identical.

We hypothesize that findings on allocentric coding in small-scale static memory-guided reaching experiments are robust, that is, they generalize to large-scale, dynamic experiments and different response modes (reaching vs. walking and placing). Therefore, we expect to find evidence for allocentric coding in both experiments. The change in the encoding task, however, should lead to a change in the weighting between egocentric and allocentric reference frames in that the interception task will lead to a mitigated use of allocentric information. In addition, it should lead to a different weighting of the landmarks in that the thrower compared to the midfield line becomes more important in the interception task.

## Materials and methods

### Experiment 1

#### Participants

Sample size was determined by means of a power analysis (G*Power 3.1.9.4; Faul et al. 2007) that resulted in a required sample of 15 participants for a power of 0.80 on detecting the allocentric weight effect (for *t*-tests against zero, with an alpha error set to 0.05). The effect size d (Cohen 1988) was obtained from previous literature and was set to 0.80. Twenty students of the Justus Liebig University Giessen were recruited via university email. The students provided informed consent and received either course credit or financial compensation. We excluded the first two participants due to technical errors during the experiment. We used the graded circle test of the Stereo Fly Test battery (Stereo Optical Co., Inc., Chicago, IL, USA) to test stereopsis. Following the test, we excluded one participant due to insufficient stereopsis leading to a final sample of 17 students (6 female; *M*_age_ = 23.18, *SD*_age_ = 2.94 years). All reported normal or corrected-to-normal vision. The experimental procedures were approved by the local ethics committee of the Justus Liebig University Giessen and are in accordance with the principles of the Declaration of Helsinki (World Medical Association et al. 2013).

#### Apparatus

Participants were positioned in the middle of a 6 × 6 m area (∼19.7 × ∼19.7 ft). The position of a standard sized 5 football (Ø = 21 cm) as well as the position of the participants’ hands was tracked by means of a 28-camera optical motion capture system (VICON Vantage, Oxford, UK) at 120 Hz. The experiment was controlled in Unity3D (Unity Technologies, San Francisco, CA, USA) ran on a Dell Alienware computer with an Intel® Core™ i7-8700 processor, 16 GB RAM and a NVIDIA® GeForce® GTX™ 1080Ti graphics processing unit. The virtual environment was presented stereoscopically with an HTC Vive Pro HMD at a resolution of 1440 × 1600 pixels per eye and a refresh rate of 90 Hz.

#### Stimuli

**Fig. 1:**
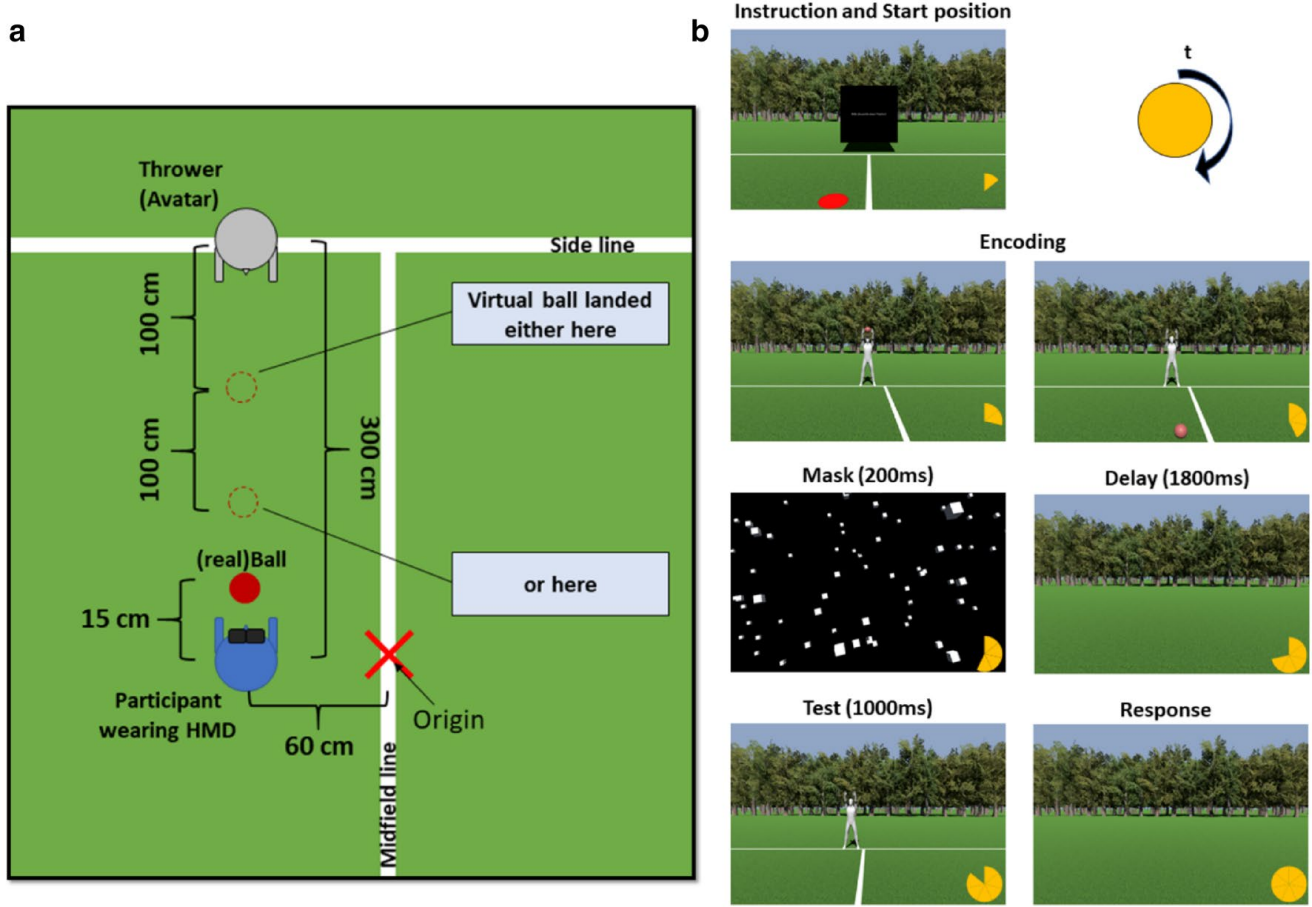
Schematic representation of the setup (**a**) and experimental procedure (**b**). The number of segments of the pie chart indicate the sequence of events

The virtual environment (Fig. 1) consisted of a soccer field on which we placed the starting point on the midfield line with participants facing the side line. We presented a 2 × 2 virtual meter (vm) floating screen at the depth of the side line to present instructions (see Fig. 1b, first row).

Participants were initially positioned 3 vm before the sideline inside the field. The radius of the ball was set to 0.21 vm. The ball was thrown such that it landed either 1 vm in front of the avatar or 1 vm in front of the participant.

The starting position of each trial was visualized by a red circle (radius of 0.25 vm) on the ground and a corresponding circle (radius of 0.25 vm) that represented the real-time position of the participants. This helped us to guide the participants to the correct starting position. The distance between the centers of both circles had to be less than 0.1 vm before the start of each trial. The starting position was 3 vm before the sideline and could either be 0.60 vm to the left or to the right of the midfield line. We created the avatar using Blender (Blender Foundation, Amsterdam, Netherlands), set it to a height of 1.75 vm and placed it either 0.60 vm to the left or to the right of the midfield line at a depth of the sideline.

To indicate the remembered target position, the experimenter placed a real ball 0.15 m in front of the participants so that they could easily find and pick up the ball when a response was prompted. The ball was made visible during the response phase. Since it was tracked by the motion capture system, the virtual ball was visible at the same position of the real ball.

Further, a mask and delay scene was created. The mask scene consisted of 800 gray cubes (0.30 vm side length) where each of them was rendered at a random angle and placed randomly covering the participant’s field of view. The delay scene consisted of the soccer field without the midfield line, thrower, and ball (i.e., grass and trees in the background).

#### Design and procedure

The experimental design (2 × 2) aimed to investigate the combination of the factor Ball Position (closer to the thrower vs. closer to the participant) and Shifted Object (thrower vs. line) resulting in four combinations. To calculate the effect of Shifted Object (for details see next section), it was necessary to measure these four combinations at least twice: once without a landmark shift (baseline) and once with a lateral landmark shift. However, to counteract spatial biases, we collected these 4 combinations three times (i.e., baseline, left shift, right shift) resulting in 12 necessary measurements. To retain test power and counteract data loss, these 12 measurements were repeated 8 times resulting in 96 trials. Within these 96 trials, we pseudo-randomly varied the starting position of both the thrower and the participant that were placed either left or right to the midline. The shifts of thrower/line were set to 0.175 vm plus noise drawn from a uniform distribution ranging from  − 0.025 to 0.025 vm. All trials were presented in a random order for each participant.

In a typical trial (Fig. 1b), participants were instructed to move to the defined starting position. Once confirmed, the encoding sequence (approximately 2100 ms) started with the avatar throwing the ball in the direction of the participant who was instructed to remember where it landed. Immediately after the ball touched the ground, the mask scene appeared (200 ms), followed by a delay (1800 ms) and a test scene (1000 ms), which contained the scene as in the encoding sequence, but without the ball and with either the line or the thrower subtly shifted or not (baseline trials). Following the test scene, participants were faced with the response scene which was similar to the delay scene. A virtual ball appeared in front of the participants and they were asked to pick up the real ball and place it at the position where they remembered the ball had landed. After participants placed the ball, they stepped back and the experimenter confirmed the positioning with a key press.

#### Data reduction and statistical analyses

The data reduction and statistical analyses were conducted in Python 3.7 and R 3.6.1. We obtained 12 measurements for each participant that were repeated eight times (= 96 trials per participant). In total, data from 17 participants were collected resulting in 1632 trials entered in data preprocessing. From these trials, we had to remove 29 trials (1.78%) because of errors during data collection. We averaged the data of each combination and calculated the lateral positioning error relative to baseline (no-shift condition) for each condition resulting in 816 data points. We then removed trials in which the lateral positioning errors exceeded 2 *SD* in Euclidean distance from group means for each condition. This affected 25 data points (3.06%) resulting in 791 values that we used for the next step. Note that the discarded samples are within the typical range of memory-guided reaching tasks (e.g., Camors et al. 2015; Van Pelt and Medendorp 2006). To show whether participants encoded the ball relative to the line or the thrower, i.e., in an allocentric reference frame, we calculate allocentric weights for both following the calculation in Karimpur et al. (2019). To this end, we simply divided the lateral positioning error by the lateral displacement of the shifted objects resulting in our key variable: the allocentric weight. To give two examples: when landmarks are shifted by 5 cm to the left and the reaching end point systematically deviates about 2.5 cm in the direction of the landmark shift, the allocentric weight is  − 2.5 ÷ − 5 = 0.5. Similarly, when landmarks are shifted by 8 cm to the right and the reaching end point systematically deviates about 4 cm to the right, the allocentric weight is 4 ÷ 8 = 0.5 as well. The idea is that if participants encoded the ball relative to the shifted object, their lateral positioning error would be biased in the direction of object shift.

For statistical analyses, we used linear mixed-effects modeling (LMM) provided with the lme4 and lmerTest library in R (Bates et al. 2015; Kuznetsova et al. 2017) to test for an effect of Shifted Object and Ball Position, both within-subject factors. Such models are well suited for our study design (Kliegl et al. 2011), as they allow us to define participants as a random factor and deal with uneven distributions in case of data loss (e.g., due to trial exclusion), thus increasing statistical power. We fitted the model by restricted maximum likelihood (REML) which is less biased for the estimation of variance components in contrast to maximum likelihood estimators. Together with the model fit, we report Satterthwaite degrees of freedom (Giesbrecht and Burns 1985; Satterthwaite 1946). We used a Shapiro–Wilk test to ensure normality of the residuals. We conducted pairwise comparisons (paired sample *t-*tests) following the LMM. To retain statistical power in case of missing pairs, we used multiple imputation (Azur et al. 2011; Buuren and Groothuis-Oudshoorn 2011; Rubin 1987). In several studies, multiple imputation has been shown to be advantageous over list-wise deletion (associated with a loss of power) and mean imputation (associated with biased estimates) in both large and small samples (Barzi 2004; Klebanoff and Cole 2008; McNeish 2017). On average, we observed negligible changes of the mean ( – 0.004) and the standard deviation ( –  0.014).

We performed *t-*tests against zero to test whether allocentric weights were significantly different from the no-shift condition. In case of violations of the normality assumption, we used a Wilcoxon test as its non-parametric equivalent to test against zero and report it right after the results of the parametric test. The results of *t-*tests will be reported nevertheless for the sake of consistency. Whenever multiple tests (*t-*tests) were conducted, we applied Benjamini–Hochberg correction for controlling false discovery rate (Benjamini and Hochberg 1995). We report Cohen’s *d*_z_ as an effect size for the *t-*tests, i.e., the difference of the means relative to the standard deviation of the difference (Lakens 2013).

### Changes in Experiment 2

We recruited 23 participants. Two participants were excluded from the dataset because both reported a misfit of the HMD and blurred image viewing after the experiment. Further, one participant reported after the experiment to have become aware of the shifts and was therefore excluded. The sample we report here consisted of 20 students (6 male; *M*_age_ = 23.24, *SD*_age_ = 3.79 years). All were tested for sufficient stereovision. Recruitment, compensation and ethical approval were the same as in Experiment 1.

The important change to Experiment 1 was that the task participants had to perform in the encoding phase. Participants were instructed to intercept the ball with their dominant foot right before the ball would touch the ground. To ensure that participants would not have to leave their starting position by running to the ball or doing a side step, we refrained from varying throwing distances and did not let the thrower stand on the opposite side of the field. Instead, the thrower was always facing the participant (left or right of the line) and the ball was always thrown 0.70 vm in front of the participants (~ closer to the participant in Experiment 1). Ball Position was therefore not varied and only Shifted Object was tested, resulting in two conditions (thrower vs. line) relevant for statistical analysis. Similar to the previous experiment, we measured these two conditions three times: once without a shift (baseline) and once with a lateral shift to the left and right, respectively, resulting in six measurements. To counteract data loss and prevent spatial biases, these six measurements were repeated ten times (60 trials in total). In half of the trials, the starting positions of the participant and the thrower were on the left of the line and in the other half of the trials on the right, following a random order for each participant. The number of repetitions took into account the duration of the experiment and the fact that it would have been too physically demanding for our participants to intercept the ball for more than an hour. No further changes were implemented with respect to the stimuli and procedure except for when participants moved too early (early lunges) or too late (ball touched the ground). In such a case, the trial was marked as failed and the next trial started.

Data reduction and statistical analyses were the same as in Experiment 1. We collected the six measurements for each participant that were repeated ten times (= 60 trials per participant) to counteract data loss. In total, data from 20 participants resulting in 1200 trials entered data preprocessing. From these trials, we had to remove 219 trials because participants moved too early or were not able to intercept the ball (18.25%). This number of failed trials was expected especially with physically demanding tasks and was only about the size of two (of ten) repetitions. With respect to the reliability of our dataset, it could be argued that the task was too difficult, resulting in a significant impact on the data. However, we believe that the complexity of the task was well chosen as participants had to highly engage in the action task to successfully intercept the ball, leading to a clearly different task during encoding than in Experiment 1. In addition, participants had to perform ten repetitions, so that the loss of 18% data still results in valid data.

After averaging we obtained 239 data points (120 for measurements on the left side of the midfield line, 119 for measurements on the right of the midfield line) for which we calculated the lateral positioning error. We removed 16 data points (6.69%) due to the 2 *SD* criterion described above, resulting in 223 values that we used to calculate the allocentric weights.

To detect an effect of Shifted Object (within-subject) and Task (between-subject), we combined the relevant subset of the data of Experiment 1 with the data of Experiment 2. The ‘relevant subset’ refers to the data from trials in which participants were facing the thrower and the ball landed close to them. Before fitting another LMM, we used a Yeo–Johnson transformation (Yeo and Johnson 2000) to reach normality of the residuals. Unlike typical Box–Cox transformations (Sokal and Rohlf 1995), this allowed the use of negative values. This was important because otherwise we would have cut out negative allocentric weights, thus, artificially pushing our results in the direction of our hypothesis (i.e., the allocentric weight effect). In the results section, we depict untransformed data for ease of interpretation. The average change caused by multiple imputation was  − 0.0035 for the mean and  – 0.0102 for the standard deviation. For the pairwise comparisons following the model fit, we used paired *t-*tests or independent *t-*test (Welch’s) when applicable. When normality assumptions were not met, we used Wilcoxon test or Mann–Whitney rank test depending on whether they belonged to the same sample or not and report it with the results of the parametric tests. We report Cohen’s *d*_z_ as an effect size for paired sample comparisons (as in Exp. 1) and *d*_s_ for independent samples, i.e., the difference of the means relative to the pooled standard deviation.

## Results

### Experiment 1

Experiment 1 aimed to investigate whether previous findings on allocentric coding for reaching generalize to large-scale and dynamic environments. To test this, we created a throw-in situation on a soccer field in virtual reality. The soccer field provided a reasonable task setting for placing contextual cues (midfield line and thrower) and using a dynamic action object (thrown ball).

**Fig. 2:**
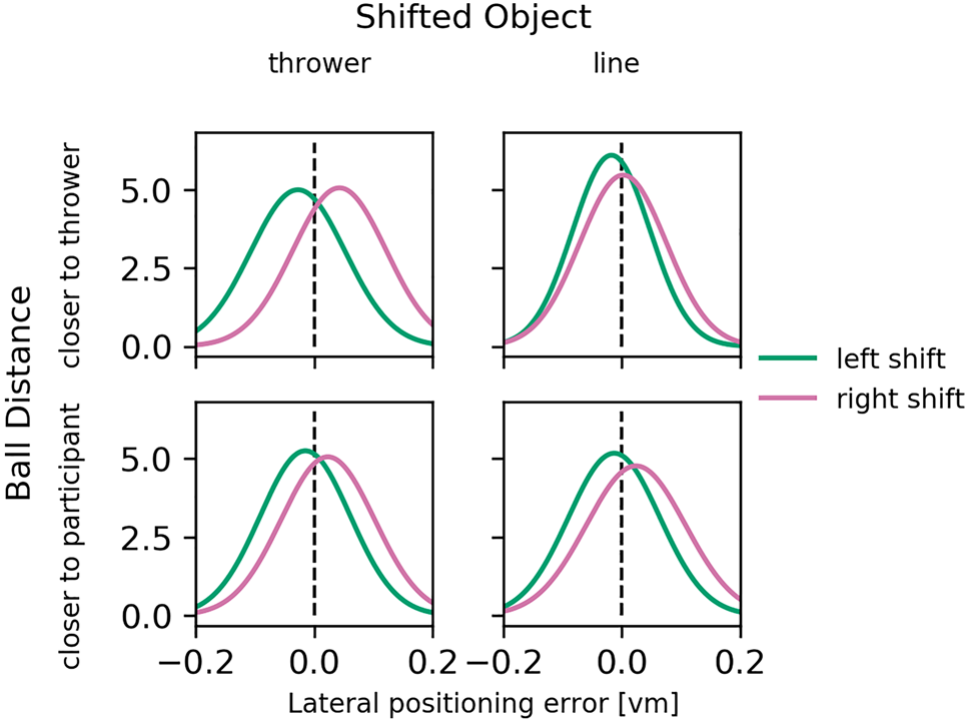
Probability density functions fitted on the lateral positioning errors (vm: virtual meters). Each plot contains 1000 sample points

A first descriptive look at Gaussian probability density functions that were based on the mean and standard deviation of the lateral positioning errors for each combination (Fig. 2) already indicates that the positioning of the ball was biased in the direction of the object shift. This was especially the case for conditions in which the thrower had been shifted.

To test whether an object shift significantly impacted the positioning, we performed *t-*tests for allocentric weights against zero (no-shift condition) for each combination of Shifted Object (line vs thrower) and Ball Position (closer to thrower vs closer to observer). All conditions yielded significant results [thrower shifted and ball closer to the thrower: *t*(15) = 5.099, *p* < 0.001, *d*_z_ = 1.275, *W* = 0, *p*_Wilcoxon_ < 0.001; thrower shifted and ball closer to the participant: *t*(15) = 3.411, *p* = 0.004, *d*_z_ = 0.853; line shifted and ball closer to the thrower: *t*(15) = 2.268, *p* = 0.039, *d*_z_ = 0.567; line shifted and ball closer to the participant: *t*(15) = 4.040, *p* = 0.001, *d*_z_ = 1.010].

We did not find a significant effect of Ball Position, but obtained a significant result for the effect of Shifted Object and for the interaction between Shifted Object and Ball Position (Table 1). Only when the ball landed in proximity of the thrower, we observed significantly higher allocentric weights when the thrower compared to the midfield line was shifted, *t*(15) = 3.580, *p* = 0.003, *d*_z_ = 0.895. We further found higher allocentric weights for shifts of the midfield line when the ball landed closer to the participant as opposed to when the ball landed closer to the thrower, *t*(15) = 2.745, *p* = 0.015, *d*_z_ = 0.686. Further tests did not survive correction for multiple testing or were not significant in the first place.

**Table 1:** Results of a linear mixed-effects model for allocentric weights

	Coef	Std. Err	*d*f	*t*	*p*	95% CI
Intercept	0.133	0.025	15	5.335	< 0.01	[ 0.083, 0.183]
Ball position	− 0.008	0.014	45	− 0.571	0.571	[− 0.036, 0.020]
Shifted object	0.040	0.014	45	2.790	0.008	[ 0.012, 0.068]
Interaction	0.035	0.014	45	2.405	0.020	[ 0.007, 0.063]

**Fig. 3:**
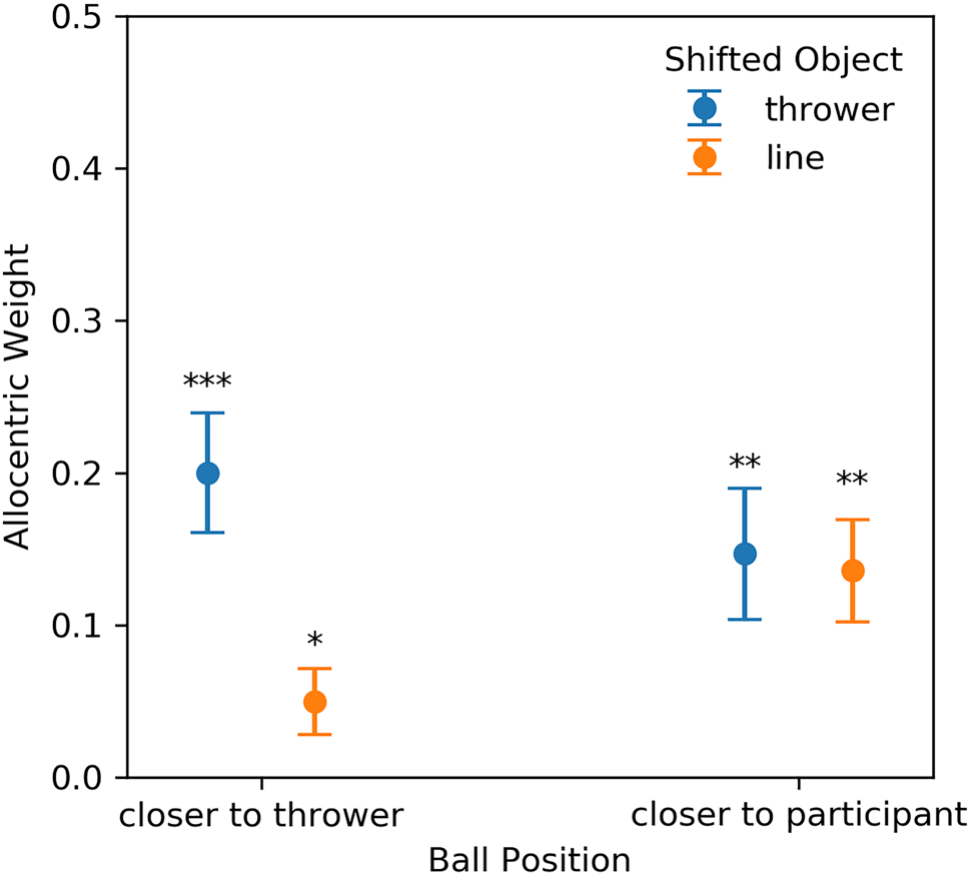
Results of Experiment 1 showing allocentric weights as a function of Shifted Object and Ball Position. Error bars represent the SEM. *p < 0.05, **p < 0.01, ***p < 0.001 for *t*-tests against zero

Our results (Fig. 3) demonstrate that participants use both the thrower and the midfield line as allocentric cues, similar to the allocentric effects reported in the previous experiments (e.g., Klinghammer et al. 2015, 2017). However, in conditions in which the ball landed close to the thrower, participants did not make significant use of the line but showed a preference to encode the ball relative to the thrower. Overall, our findings support the use of allocentric information for action and generalize previous results to large-scale and dynamic environments.

### Experiment 2

We confirmed that even in a dynamic scene without a prolonged encoding period, participants use allocentric information such as the thrower and the midfield line. In Experiment 2, we aim to answer whether a different task determines the use of allocentric information during spatial encoding for action. To test this, participants were asked to intercept the thrown ball with their dominant foot, i.e., the encoding of the ball’s location was coupled with an action. On the one hand, performing an action requires a spatial coordinate transformation to an egocentric reference frame (see Crawford et al. 2011) that should facilitate egocentric coding of the ball’s landing position. If the ball’s landing position is predominantly encoded in an egocentric reference frame, we would expect generally lower allocentric weights compared to the corresponding data of Experiment 1. On the other hand, successfully intercepting the ball could increase the importance of the thrower and therefore the usage of the thrower as a landmark that should result in higher allocentric weights for the thrower than the midfield line compared to the corresponding data of Experiment 1.

**Fig. 4:**
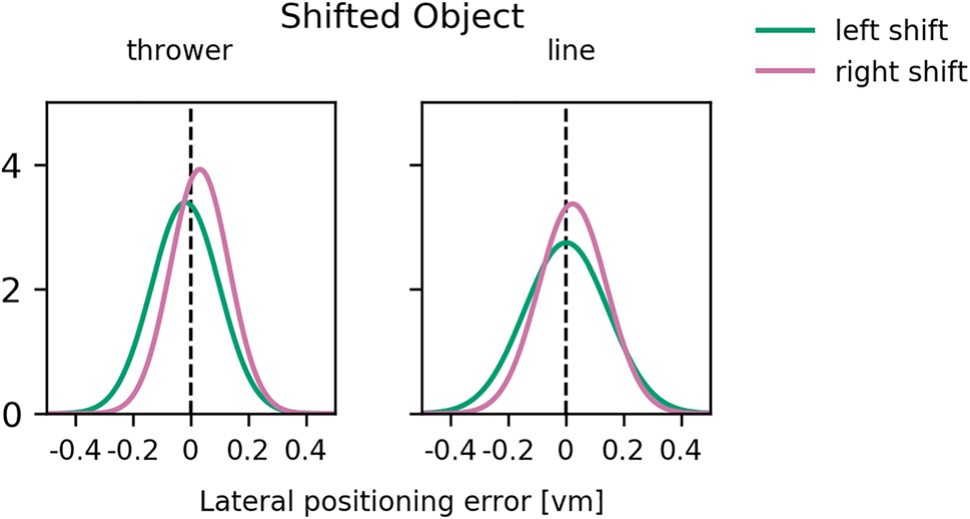
Probability density functions (PDF) for both Shifted Objects and both shift directions. The PDFs were fitted on the lateral positioning errors. Each plot contains 1000 sample points

**Fig. 5:**
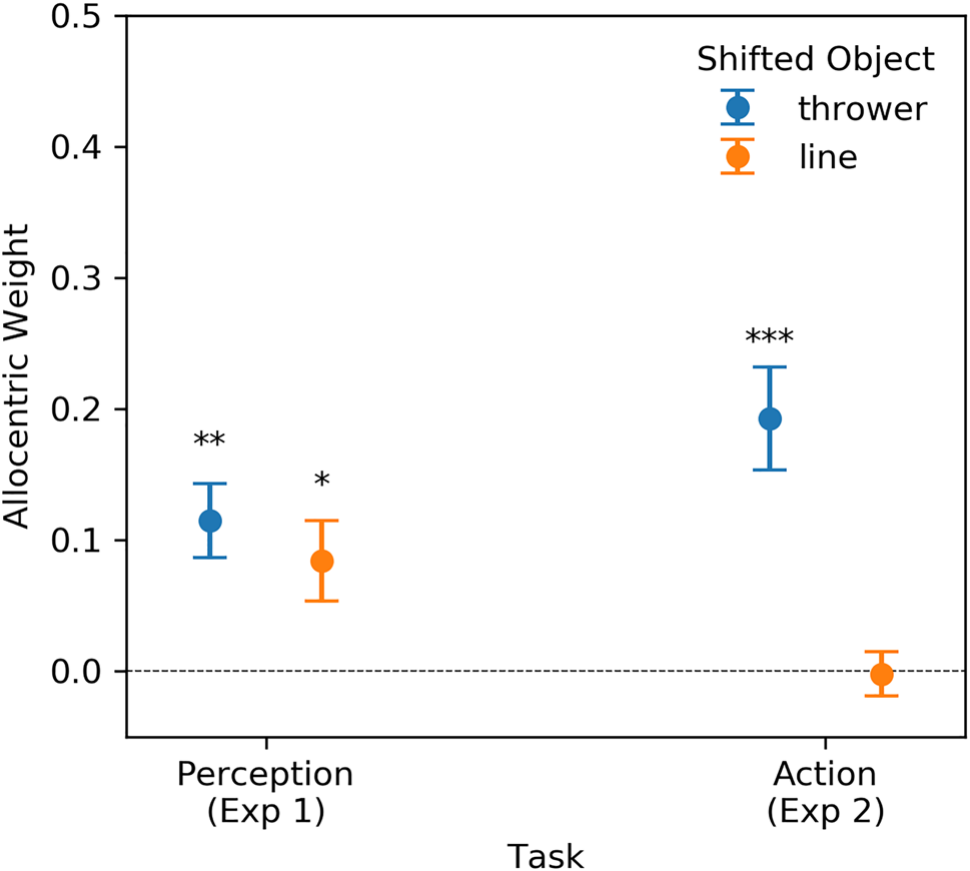
Comparison of allocentric weights as a function of Shifted Object between Experiment 2 and the relevant subset of Experiment 1. The error bars represent the standard error of mean. *p < 0.05, **p < 0.01, ***p < 0.001 for tests against zero

To ensure that participants could stay at their starting position, we refrained from using two throwing distances. This leaves us with the factor Shifted Object (thrower vs line), for which the results are depicted in Figs. 4 and 5.

On a descriptive level, the fitted distribution of lateral positioning errors indicated a positioning bias in the direction of the object shift for conditions in which the thrower had been shifted. This is also reflected in the allocentric weights that are significantly above zero [*t*(17) = 4.912, *p* < 0.001, *d*_z_ = 1.158] which was not the case when the line was shifted [*t*(17) =  − 0.134, *p* = 0.895, *d*_z_ = 0.032]. The results of the LMM (Table 2) based on the merged data reveal a significant effect of Shifted Object and a significant interaction between Shifted Object and Task.

**Table 2:** Results of a linear mixed-effects model for allocentric weights of the merged data

	Coef	Std. Err	*d*f	*t*	*p*	95% CI
Intercept	0.070	0.012	31	6.007	< 0.001	[ 0.047, 0.093]
Task	0.006	0.012	31	0.528	0.601	[ − 0.017, 0.029]
Shifted object	0.043	0.010	31	4.421	< 0 .001	[ 0.024, 0.062]
Interaction	− 0.030	0.010	31	− 3.058	0.005	[ − 0.049, − 0.011]

Pairwise comparisons show that, in Experiment 2, allocentric weights were lower for the shift of the midfield line (*M* = 0.00, *SEM* = 0.02) than for the shift of the thrower [*M* = 0.19, *SEM* = 0.04, *t*(17) = 4.542, *p* < 0.001, *d*_z_ = 1.071, *W*_Wilcoxon_ = 3, *p*_Wilcoxon_ < 0.001]. There was no such difference in Experiment 1 [thrower: *M* = 0.12, *SEM* = 0.03, line: *M* = 0.08, *SEM* = 0.03, *t*(14) = 0.920, *p* = 0.373, *d*_z_ = 0.238]. Both levels of Shifted Object in Experiment 1 revealed higher allocentric weights than the shift of the line in Experiment 2 [thrower: *t*(31) = 3.528, *p* = 0.002, *d*_s_ = 1.333; line: *t*(31) = 2.451, *p* = 0.023, *d*_s_ = 0.935]. Shifts of the thrower in Experiment 2 revealed higher allocentric weights than the shift of the line in Experiment 1 [*t*(31) = 2.184, *p* = 0.037, *d*_s_ = 0.800, *U*_Mann–Whitney-*U*_ = 79, *p*_Mann-Whitney-*U*_ = 0.022].

## Discussion

In two virtual reality experiments, we found evidence for allocentric coding of action targets in large-scale and dynamic environments. We report increased allocentric weights for landmark shifts of the thrower as well as shifts of the midfield line when encoding is merely visual (Experiment 1) and remarkably increased allocentric weights for shifts of the thrower compared to shifts of the midfield line, which did not yield an effect, when encoding required to intercept the thrower’s ball with the foot (Experiment 2). These results support previous research on allocentric coding using static scenes that showed that humans encode objects for action to some extent relative to contextual cues in the environment. The allocentric weights we found here are quite comparable to previous virtual reality studies varying between approximately 0.10 and 0.50 (Karimpur et al. 2019; Klinghammer et al. 2016). More importantly, these numbers were found despite the change in response mode. In contrast to previous experiments on memory-guided reaching, we asked participants to grab a ball (tracked via motion capture) with two hands and walk to and place it as accurately as possible to where they believe the ball had landed. This response mode required sequential, large-scale movements including different effector systems. Our findings support the idea that in such tasks, we do not simply encode a point in space as a point on our retina, but rather form stable spatial representations that can be used to successfully perform goal-directed actions.

### The encoding task determines how allocentric information is used

In the second experiment, we examined whether the nature of the task during spatial encoding has an impact on the use of allocentric information. We know from previous work on allocentric coding of reach targets that task-relevant objects are predominantly used as allocentric cues, whereas task-irrelevant ones are mostly ignored (Klinghammer et al. 2015). Here, we changed the encoding task from purely visual to perception for action by asking participants to intercept a ball with their foot. We found no effect for the shift of the midfield line, but a clear effect for the shift of the thrower, suggesting that participants still relied on allocentric cues but that some cues were more effective than others. These results support the hypothesis that the interception task increased the importance of the thrower and therefore the usage of the thrower as a task-relevant landmark. We did not find evidence for the hypothesis that participants rely generally less strongly on allocentric information when asked to encode the target location by performing a movement. This hypothesis was based on previous findings suggesting that performing an action requires the transformation of spatial information into an egocentric reference frame (Crawford et al. 2011), and as a consequence decreasing the weighting of allocentric cues. Experiments using reaching movements showed that this transformation occurs as soon as the reach direction can be calculated, i.e., at the first possible opportunity (Chen et al. 2018). For example, if an action such as intercepting the ball is required, the transformation already occurs at the encoding stage while it may occur later if an action is not required until the response, as in Experiment 1. One could therefore argue that the task in Experiment 2 might have facilitated an early use of an egocentric reference frame. However, Chen and colleagues (2018) also emphasize that an ‘Allo-to-Ego’ transformation does not require the initial allocentric information to be dumped, but instead the allocentric information may persist in parallel to egocentric information. This is in line with our results and suggests that participants encoded the location of the ball to a significant amount relative to the thrower, i.e., in an allocentric reference frame.

Our results could be interpreted in the framework of the dual streams for perception and action (Goodale and Milner 1992), suggesting that visual information is processed by two separate streams. The ventral stream processes visual information in the inferotemporal cortex for perception and is associated with the use of allocentric information. The dorsal stream, running through the occipital–parietal areas, uses visual information to guide actions and is associated with the use of egocentric information (Chen et al. 2011; Schenk 2006; Westwood and Goodale 2003). More recent studies on the neural substrates of allocentric coding are in line with this idea (Chen et al. 2014, 2018). These studies describe, for example, the inferior occipital gyrus as a common hub for visuospatial memory, but also dedicated areas such as the inferior temporal gyrus for allocentric memory. In Experiment 2, participants used visual information to guide their action (i.e., to intercept) during encoding, but had to access the relevant representation after a delay to give their response. We believe that another reason why allocentric information played such an important role here is that different representations were used for encoding and response. The idea of different representations for immediate and delayed actions has been discussed for decades (e.g., Goodale et al. 2004; Rossetti 1998), reviewed by Bruno (2008), and more recently by Schenk and Hesse (2018) who suggest the dorsal amnesia hypothesis. According to the hypothesis, the perceptual or ventral stream maintains visual information while the visuomotor system or dorsal stream depends on real-time visual information. Actions that are based on memory representations are therefore mainly guided by the ventral system, while immediate actions are mainly guided by the dorsal system. Intercepting the ball requires using the visuomotor system. However, according to the dorsal amnesia hypothesis, it does not matter whether we encode the position perceptually or by performing an action. Because of the delayed nature of the task (place the ball at the remembered location), our memorized visual information will be mostly allocentric. It comes therefore as no surprise that allocentric information was used in Experiment 2. The important role of the thrower needs to be discussed from a different perspective, though.

The results of both experiments can be reconciled by considering the reliability of each spatial cue in the context of the task at hand. In Experiment 1, the task was different, i.e., no interaction with the target object (the ball) was required. As previous studies showed that allocentric information can increase movement accuracy and precision (Krigolson et al. 2007; Krigolson and Heath 2004; Obhi and Goodale 2005), using all available environmental information (i.e., the thrower and the line) can help us to build an accurate positional estimate. In Experiment 2, a trial was only deemed as valid when participants successfully intercepted the ball right before touching the ground. The task here required using contextual cues that could allow participants to improve their prediction on the future trajectory of the ball. We believe that this information was used to get an estimate about the lateral component of the future landing position, since the ball was always thrown in a prototypical manner straight away from the thrower, i.e., the thrower might have been undoubtedly more reliable as a cue than the midfield line. During the actual movement of the ball, the contextual information (position of the thrower) may become less important for successful interception (cf., Kreyenmeier et al. 2017). The task relevance of the midfield line, in turn, was even more reduced in the interception task and thus this information was not integrated in the spatial target representation (cf., Klinghammer et al. 2015).

Studies on eye movements in ball interception tasks can also provide an interesting angle on our findings. Early work already showed that participants’ gaze does not follow the entire ball’s trajectory (Ripoll and Fleurance 1988). One marker that has been discussed was the last fixation before a ball was thrown, which was supposed to be indicative of interception success (so called “quiet eye”, Panchuk and Vickers 2006; Vickers and Adolphe 1997). Studies that looked at soccer goalkeepers were able to show that expert goalkeepers preferably fixate the legs of a player in contrast to novices who fixate the trunk and other body parts (Savelsbergh 2005; Savelsbergh 2002; for a review on expertise differences, see Mann et al. 2007). This distinction between experts and novices should not be of concern in our study, because in both cases, the thrower (or kicker) is observed. Future studies could further examine gaze behavior in such type of shift paradigm and could even manipulate the position of single body parts as part of the “shift manipulation”. While some studies looked at the role of body language (Prigent et al. 2014), others emphasized the important role of pursuit duration or accuracy (Cesqui et al. 2015; Fooken et al. 2016). Today, many studies demonstrate that our eyes follow the ball’s trajectory and make predictive saccades (close) to the predicted bounce location (Diaz et al. 2013; Hayhoe et al. 2012; Land and McLeod 2000). In our experiment, the lateral position of the thrower could be used quite early for such accurate predictions of the bounce location. By investigating gaze behavior, one could gain a deeper understanding of the strategies employed in the shift paradigm.

Gaze behavior also plays an important role when examining overt attention. It was shown that objects that are task relevant (i.e., they pose different demands to the observer) are longer and more frequently fixated (Land and Hayhoe 2001), more effectively retained in visual working memory (Maxcey-Richard and Hollingworth 2013), and more likely to be coded allocentrically (Klinghammer et al. 2015). When task demands increase, e.g., by changing or adding a task, humans allocate their attention accordingly (e.g., by saccading back and forth when needed) to successfully perform the tasks (Hayhoe and Ballard 2005). For covertly attended locations, it was shown that they are processed with higher spatial acuity at the costs of spatial acuity in unattended locations (Montagna et al. 2009). Summed up, findings on eye movements and allocation of spatial attention give potential explanations why a change in the task performed during encoding can lead to an increased importance of the thrower. The thrower was not only task relevant, but also posed increased task demands to participants because, to successfully intercept (right location at the right time), they had to pay attention to the thrower.

### Limitations

We aimed to create a large-scale experiment by using a naturalistic real-world-like scene with a realistic cover story. The soccer throw-in situation served this purpose, but came at costs of changes relative to previous studies. For example, we changed the encoding duration from self-paced to a glimpse of a second and changed the response mode from mere pointing or reaching to walking and placing the ball. Nevertheless, we found similar results which further corroborates the findings. Further differences concern the relatively low number of potential allocentric cues and the a priori information about the action target. Yet, our results replicated previous findings showing that prior knowledge about the target object attenuates allocentric coding but does not cancel it out (Lu et al. 2018). Lastly, the majority of studies in our field has been relying on upper limb movements to study the human visuomotor system. The fact that we used lower limb movements to encode the location of the action target should not be of concern. Participants used lower limb movements to perform the action during encoding, but, irrespective of the limb, the location has to be bound to a common spatial representation (for a review, see Crawford et al. 2011). Further, studies on visual illusions suggested that upper and lower limb movements are bound to a common visuomotor system (Glover and Dixon 2004). Neuroimaging studies looked at the organization of the posterior parietal cortex and the superior parietal lobule which are crucial for sensorimotor integration. In contrast to early findings that were traditionally interpreted in terms of an effector-specific organization (e.g., as reviewed by Andersen and Cui 2009), more recent work found evidence for both limb-specific and limb-unspecific motor regions (Heed et al. 2016; Medendorp and Heed 2019).

We conducted this study by means of virtual reality which inherently raises the question of ecologic validity. Early studies were able to corroborate the similarity between virtual reality and real-world findings (Laczó et al. 2012; Lloyd et al. 2009). However, in a recent review, Harris, Buckingham, Wilson and Vine (2019) discuss intriguing challenges that are relevant for virtual reality studies, especially for those focusing on the dual stream model of perception and action (Goodale and Milner 1992). One such challenge is the difference between depth information in the real world and artificial (and therefore compressed) depth information in virtual environments. Due to the lack of binocular cues, humans have to increasingly rely on monocular cues which inherently leads to an increased use of ventral stream information (Marotta et al. 1998). We already discussed how ventral stream information has often been associated with allocentric coding. In that line of thought, future studies could try to test a paradigm similar to the one we used in the real world. Of course, this poses technical challenges such as thorough experimental control that have to be overcome first. To further generalize our findings, future experiments should consider the reliability of contextual cues. This can be done, for example, by introducing movement sequences (e.g., side steps) or just visually by blurring some contextual cues.

The design of Experiment 2 was a mixed-effects design with Task (during encoding) as a between-subjects factor. Integrating an additional factor Task to Experiment 1 would have doubled the duration of the experiment. Since the response mode itself (“grab the ball, walk over and place it”) was already quite demanding, we refrained from including that in the first experiment while accepting the obvious drawbacks (e.g., individual variability). Instead, we investigated the effect of the throwing distance in Experiment 1 to gain a first insight into the role of target proximity. We ensured similarity between the stimulus configurations of both experiments by using the same virtual environment and leaving the stimuli unchanged (for details see Methods, subsection “Experiment 2”). In Experiment 1, we found differences between the landmarks when the ball landed closer to the thrower, but not when it landed closer to the observer. In Experiment 2, we kept the “closer to the observer” distance to allow successful interception without leaving the starting position. To this end, we had to slightly adjust the distance between the ball and the observer so that the ball was more easily interceptable. It is unlikely that bringing the ball approximately one foot closer resulted in an incomparable configuration.

Our sample consisted of an uneven number of female and male participants which might raise concerns. In the field of spatial navigation, it has often been reported that performance in spatial tasks differs between gender (e.g., Moffat et al. 1998; Padilla et al. 2017; Picucci et al. 2009; Saucier et al. 2002). In some cases, it would be interesting to see if such findings from navigation research generalize to the field of spatial coding for action (here: memory-guided reaching or placing tasks). To the best of our knowledge, there are no studies that found an effect of gender in the shift paradigm. This can also be corroborated with our own data from previous experiments which do not indicate a gender effect.

## Conclusion

In sum, we provide evidence that spatial representations are quite robust with regard to different encoding tasks and response mode. We demonstrate allocentric coding of target locations that had been encoded perceptually as well as by performing an action. How is observing a ball that was thrown in your direction different from catching it? The answer is that it is not really different as long as the brain deems the contextual cues to be task relevant and reliable. The reliability of each contextual cue, however, has to be evaluated in the face of the current task demand.
